# Long-range neural activity evoked by premotor cortex stimulation: a TMS/EEG co-registration study

**DOI:** 10.3389/fnhum.2013.00803

**Published:** 2013-11-25

**Authors:** Marco Zanon, Piero P. Battaglini, Joanna Jarmolowska, Gilberto Pizzolato, Pierpaolo Busan

**Affiliations:** ^1^Cognitive Neuroscience Sector, International School for Advanced Studies, SISSATrieste, Italy; ^2^Department of Life Sciences, BRAIN Center for Neuroscience, University of TriesteTrieste, Italy; ^3^Department of Medical, Surgical and Health Sciences, University of TriesteTrieste, Italy

**Keywords:** premotor cortex, propagated activity, TMS/EEG co-registration, sLORETA, TMS-evoked potentials

## Abstract

The premotor cortex is one of the fundamental structures composing the neural networks of the human brain. It is implicated in many behaviors and cognitive tasks, ranging from movement to attention and eye-related activity. Therefore, neural circuits that are related to premotor cortex have been studied to clarify their connectivity and/or role in different tasks. In the present work, we aimed to investigate the propagation of the neural activity evoked in the dorsal premotor cortex using transcranial magnetic stimulation/electroencephalography (TMS/EEG). Toward this end, interest was focused on the neural dynamics elicited in long-ranging temporal and spatial networks. Twelve healthy volunteers underwent a single-pulse TMS protocol in a resting condition with eyes closed, and the evoked activity, measured by EEG, was compared to a sham condition in a time window ranging from 45 ms to about 200 ms after TMS. Spatial and temporal investigations were carried out with sLORETA. TMS was found to induce propagation of neural activity mainly in the contralateral sensorimotor and frontal cortices, at about 130 ms after delivery of the stimulus. Different types of analyses showed propagated activity also in posterior, mainly visual, regions, in a time window between 70 and 130 ms. Finally, a likely “rebounding” activation of the sensorimotor and frontal regions, was observed in various time ranges. Taken together, the present findings further characterize the neural circuits that are driven by dorsal premotor cortex activation in healthy humans.

## Introduction

In the last decades, many studies have contributed to disentangle the anatomical and functional organization of the cortical circuitries characterizing motor structures. Most have focused on control of motor behavior (see for a brief review Rizzolatti and Luppino, 2001), and/or on goal-directed movements under visual guidance (Naranjo et al., 2007). The dorsal premotor cortex (PMd) plays a key role in these motor networks (Davare et al., 2006). It is actively involved in several functions ranging from the planning of a proper motor response to the correct allocation of attentive resources (Rushworth et al., 2003) and/or eye-related activity (Luppino and Rizzolatti, 2000; Amiez and Petrides, 2009). As a consequence, the PMd is directly or indirectly linked with a series of structures in the brain (Hagmann et al., 2008), ranging from the primary motor cortex (Matsumoto et al., 2007; Huang et al., 2009) and the supplementary motor area (Matsumoto et al., 2007) to the superior parietal cortex (Kurata, 1991; Massimini et al., 2005; Rottschy et al., 2012), parieto-occipital regions (Shipp et al., 1998; Caminiti et al., 1999), and the prefrontal cortex (Barbas and Pandya, 1987; Lu et al., 1994), in order to allow effective exchange and elaboration of information.

Taking into consideration the goal-directed movements under visual guidance, Milner and Goodale (2006) suggested the existence of different streams mediating the sensory-motor transformations necessary for visually guided movements. Signals elaborated in the primary visual cortex are sent to areas for integration with other sensory information to organize a representation of the action to be performed. The information is then transmitted to the frontal cortex, such as Brodmann area 6, which constitutes the premotor cortex in humans and successively sends motor programs to the primary motor cortex (see for a brief review Luppino and Rizzolatti, 2000; Rizzolatti and Luppino, 2001). Furthermore, the PMd can be functionally subdivided in subregions, according to the specific computation it is mainly involved in. For example, neurons involved in reaching movements have been identified in the dorsal part of the lateral premotor cortex, whereas its ventral part is likely involved in grasping movements (Rizzolatti et al., 1998; Rizzolatti and Luppino, 2001; Matsumoto et al., 2007).

Most of the knowledge about the functional and anatomical organization of PMd derives from studies on animals, and in particular on non-human primates (Rizzolatti et al., 1998), but also on electrophysiological recordings during neurosurgery, as in the case of epileptic patients (Matsumoto et al., 2003, 2007). However, the development of non-invasive neuroimaging techniques extended knowledge of PMd connectivity and functions even in humans (Picard and Strick, 2001; Massimini et al., 2005; Rottschy et al., 2012). Early electrophysiological studies on monkeys have shown that PMd neurons respond, for example, to the appearance of visual signals and discharge during the preparation and execution of movements under visual guidance (Hoshi and Tanji, 2006). The PMd is also activated by viewing an object that has motor valence, even in the absence of a subsequent movement (Grafton et al., 1997). Moreover, it is also involved in motor attention and motor selection (Rushworth et al., 2003). Some of these findings have also been observed in humans (Davare et al., 2006) and it is now clear that the PMd is part of a fronto-parietal circuit for goal-directed movements, where it plays a key role in the planning aspects of motor commands (Luppino and Rizzolatti, 2000; Rizzolatti and Luppino, 2001; Davare et al., 2006; Milner and Goodale, 2006; Naranjo et al., 2007; Busan et al., 2009a).

The PMd organizes and selects appropriate and effective motor commands, on the basis of representations, provided by the parietal regions, and intentions, elaborated in the prefrontal areas (Tanne et al., 1995; Matelli and Luppino, 2001; Galletti et al., 2003). The role of the PMd as an integration center is also supported by studies that have shown a functional gradient of neuronal projections with its rostral part mainly connected to prefrontal regions, and caudal portions sending projections mainly to the primary motor cortex and spinal cord. This organization has led to the hypothesis that the former are more likely involved in higher-level, cognitive aspects of behavior preparation, whereas the latter are probably involved in less complex functions that are more related to motor execution (Picard and Strick, 2001; Matsumoto et al., 2003). The PMd seems to be involved in the control of eye movements and in the control of eye-related neural activity or in specific tasks that require eye-hand coordination (Luppino and Rizzolatti, 2000; Amiez and Petrides, 2009).

Due to the functional relationship between the parietal and frontal areas, it has been suggested that the flow of information is not unidirectional from the former to the latter, but that it can re-enter the parietal cortex through fronto-parietal connections. This reciprocity of cortico-cortical connections implies that coding of information cannot be regarded as a serial sequence of transformations, each performed by a given cortical area, but rather as a recursive process that can also involve relatively remote regions (Paus et al., 1997; Battaglia-Mayer et al., 1998; Massimini et al., 2005). In spite of all this information, effective and functional connections of PMd in humans are still far from being fully understood.

Different techniques to investigate brain connectivity have been developed during recent years, and among these, the combination of transcranial magnetic stimulation (TMS) with electroencephalography (EEG) acquisition (TMS/EEG) has been demonstrated to be a useful approach. It allows the study of the propagation of neural activity from the stimulated cerebral area to other brain regions, providing a new way to look for their connections (e.g., Ilmoniemi et al., 1997). In fact, thanks to the optimal temporal resolution offered by EEG and the reconstruction of the EEG neural sources (Michel et al., 2004), it is possible to successfully characterize the neural temporal dynamics of events evoked by TMS (TMS-evoked potentials, TEPs; e.g., Paus et al., 2001; Bonato et al., 2006).

Although the mechanisms underlying TEPs are not completely understood, they might provide useful information with respect to brain functions and networks (Massimini et al., 2005; Komssi and Kahkonen, 2006; Miniussi and Thut, 2010; Frantseva et al., 2012; Manganotti and Del Felice, 2013). Furthermore, the development of improved methods to obtain a reliable EEG source estimation allows adding spatial information on the evoked neural activity (e.g., Ilmoniemi et al., 1997; Pascual-Marqui, 2002).

TMS/EEG is usually performed by using an “inductive” approach (Miniussi and Thut, 2010), and a long-scale network of neuronal connections has been shown to be engaged when TMS-related activation spreads from the stimulation site toward different brain regions, an activity that can last even hundreds of milliseconds (e.g., Ilmoniemi and Karhu, 2008). The properties of the TMS-evoked responses have been shown to depend on a series of parameters, such as the stimulus intensity (Casarotto et al., 2010). In this regard, while the initial part of the TEP can reflect the “reactivity” of the stimulated cortex, the later spatio-temporal propagation of the electrical activity toward different brain regions might unravel the presence of intra- and/or inter-hemispheric cortico-cortical connections as well as intermediate links, such as subcortical structures.

In an effort to better characterize PMd connectivity, we used TMS/EEG co-registration to investigate the cortico-cortical long range connections and activity propagation pathways. We focused on the stimulation of the left hemisphere, usually viewed as the dominant one in right-handed people (e.g., Iacoboni, 2006; Vingerhoets et al., 2013). In this sense, the left hemisphere seems to play a special role also in organizing movements during visually-guided praxis (e.g., Goodale, 1988; Janssen et al., 2011).

## Materials and methods

### Subjects and transcranial magnetic stimulation

**Figure 1:**
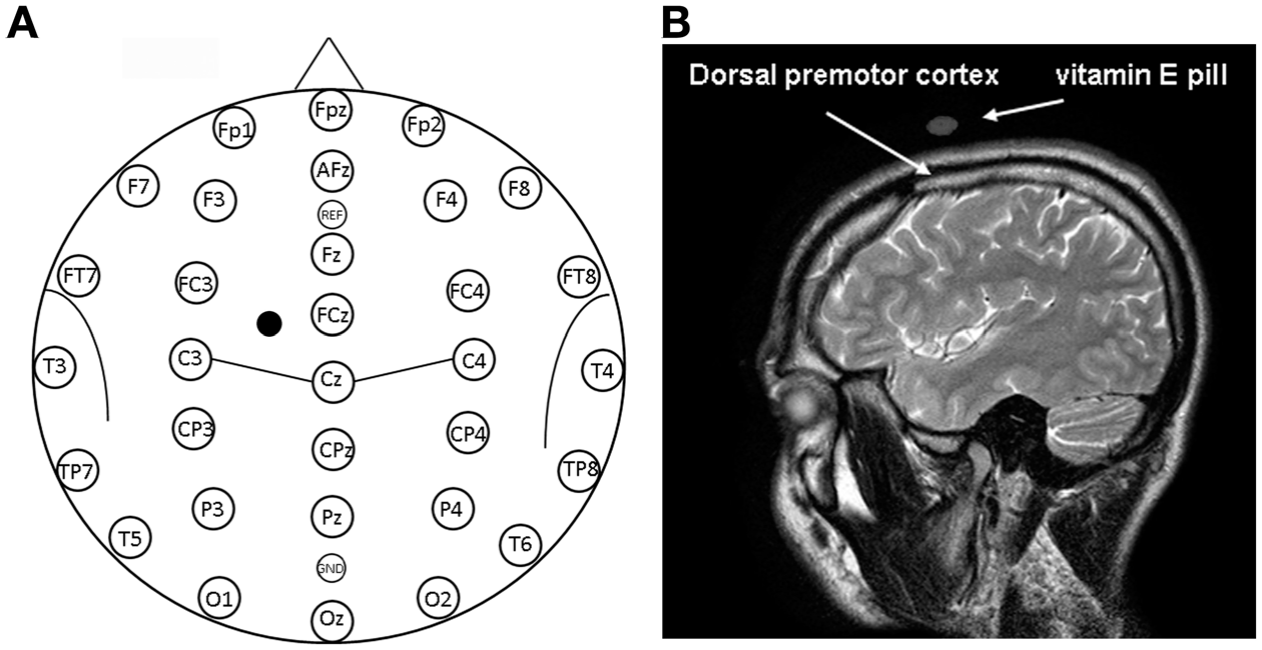
**(A)** Location of EEG electrodes and point of stimulation. Exemplification of positioning of EEG electrodes used for recordings is reported on a model also indicating some of the main brain sulci. The point on the scalp where TMS was applied is indicated by a mark. **(B)** Structural magnetic resonance indicating the point of stimulation. Anatomical magnetic resonance acquisition performed in a prototypical subject. The point of stimulation is indicated by the positioning of the vitamin E pill. The positioning of the dorsal premotor cortex is also indicated on the basis of Duvernoy (1999).

Twelve healthy subjects (7 males and 5 females, age range 22–26 years, mean age 23.4 years, *SD* = 1.2) underwent single-pulse TMS (Medtronic MagPro R30) applied on a scalp position that putatively allowed the stimulation of the dorsal premotor cortex (see Figure 1). All subjects were right-handed as confirmed by a dedicated questionnaire (Edinburgh Inventory; Oldfield, 1971). Participants gave written informed consent after receiving exhaustive information about all procedures, in compliance with the Declaration of Helsinki. Favorable judgment of the Ethics Committee of the University of Trieste was obtained. Participants could leave the study at any time, although all completed the experiments.

The stimulated scalp position was determined using an adapted EEG coordinate system (see Herwig et al., 2003; Okamoto et al., 2004; Jurcak et al., 2007) and a probabilistic method (Steinsträter et al., 2002; http://wwwneuro03.uni-muenster.de/ger/t2tconv/). TMS was delivered on a scalp location that corresponded to a position situated 10% of the biauricolar distance to the left of the vertex and 7.5% ahead the nasion-inion distance (see Figure 1A). A very rough estimation, in MNI (Montreal Neurological Institute) coordinates, of the center of the stimulated region was: *x* = −30, *y* = 10, *z* = 65 (best match: Brodmann Area—BA—6). The resulting point of stimulation was marked on the EEG cap.

For each participant, TMS was delivered through a figure-of-eight coil (diameter of each wing about 7 cm), oriented tangentially to the scalp (single pulse stimulation; biphasic waves; pulse duration: 280 μsec). The coil was secured on the scalp by hand and its position was continuously visually checked and readjusted if necessary. The coil was maintained with a 45° orientation with respect to the inter-hemispheric fissure with the handle pointing downward and backward. The subject's head was not restrained, although participants were asked to maintain a stable position for the entire experiment with their chin backed on a metal structure. The stimulation coil was also maintained in the same position when sham TMS was delivered.

### Pre-experimental procedures

Before each experiment, the optimal cortical point for activating the first dorsal interosseous muscle (FDI) of the right hand was individuated and, successively, resting motor threshold (RMT) was measured as the stimulus intensity triggering at least a 50 μ V response on electromyography traces (EMG; band pass filtering 20–2000 Hz) in half of the stimulations (Rossini et al., 1994). A tendon belly montage was used by applying surface Ag/AgCl electrodes. During experimental sessions, the intensity of TMS was set at 110% RMT to limit current diffusion to neighboring areas, such as the primary motor cortex. To check for unintended current diffusion, the selected stimulation point on PMd was stimulated immediately after the evaluation of RMT, before the beginning of the experiment. Specifically, muscular responses on different right hand and right arm muscles, detected by EMG (band pass filtering 5–2000 Hz), were considered as unwanted activation of the primary motor cortex at the individuated experimental intensity. On the same line, it was verified that stimulation of the premotor cortex did not evoke evident facial muscular artifacts (e.g., Julkunen et al., 2008; Mütanen et al., 2013). When a muscular response was highlighted, one of the following operations or combination was used to reduce these muscular activations until no response was evident: a) the stimulation intensity was slightly dampened; b) the coil position was slightly moved anteriorly; c) the coil was slightly rotated from its original position. Some of these suggestions have been already shown to be effective in reducing muscular artifacts (Ilmoniemi and Karhu, 2008; Ilmoniemi and Kicic, 2010). As a consequence, for each subject we found the methodological solution that minimized variability with respect to the original setting in terms of stimulation intensity, stimulated scalp position, and coil position. It is evident that the application of these procedures could make individuation of the premotor cortex a little bit more uncertain, but they also allowed minimizing possible contamination from neighboring neural regions, especially the primary motor cortex, reducing, for example, artifacts related to sensory feedbacks obtained after distal muscular activations.

### TMS/EEG experimental setting

Subjects were asked to sit with eyes closed for the entire duration of stimulation blocks (real and sham TMS sessions) to reduce ocular artifacts.

The experiment consisted of three blocks of 65 real magnetic stimuli (real TMS) and three blocks of 65 sham stimuli (sham TMS), interleaving one real TMS block and one sham TMS block. The starting block (real or sham TMS) was randomly defined. During stimulations, EMG was constantly checked to verify that the stimuli did not evoke any muscular response that could interfere with EEG recorded potentials, such as that evoked by direct motor activation or somatosensory feedbacks successive to distal muscular activations. For this purpose, right hand muscles (first dorsal interosseous muscle, abductor digiti minimi muscle and opponens pollicis brevis muscle) were routinely monitored as well as right arm biceps brachii muscle, right arm deltoid muscle and/or right arm flexor and extensor muscles. Monitoring was performed by applying surface Ag/AgCl electrodes on the targeted muscles and using a band-pass filtering of 5–2000 Hz. Trials showing an evident motor response were discarded from successive analyses.

Sham TMS was performed by applying the coil on the scalp in the same manner as real TMS condition, and using the same intensity of stimulation. In this condition, however, a 3-cm-thick block of wood was placed between the coil and scalp to reduce the intensity of the magnetic field that reached the scalp. Both in real and sham conditions, about 0.5 cm of foam was applied between the scalp and coil to limit the somatic sensation specifically related to TMS stimulation; subjects wore earplugs to reduce acoustic stimulation. In this way, sham TMS allowed to control for the acoustic activation related to magnetic stimulation (Nikouline et al., 1999), while a reliable control for the somatic sensation of TMS is not so simple to obtain. Safety guidelines for TMS were always taken into consideration (e.g., Wassermann, 1998; Rossi et al., 2009).

### EEG data

**Figure 2:**
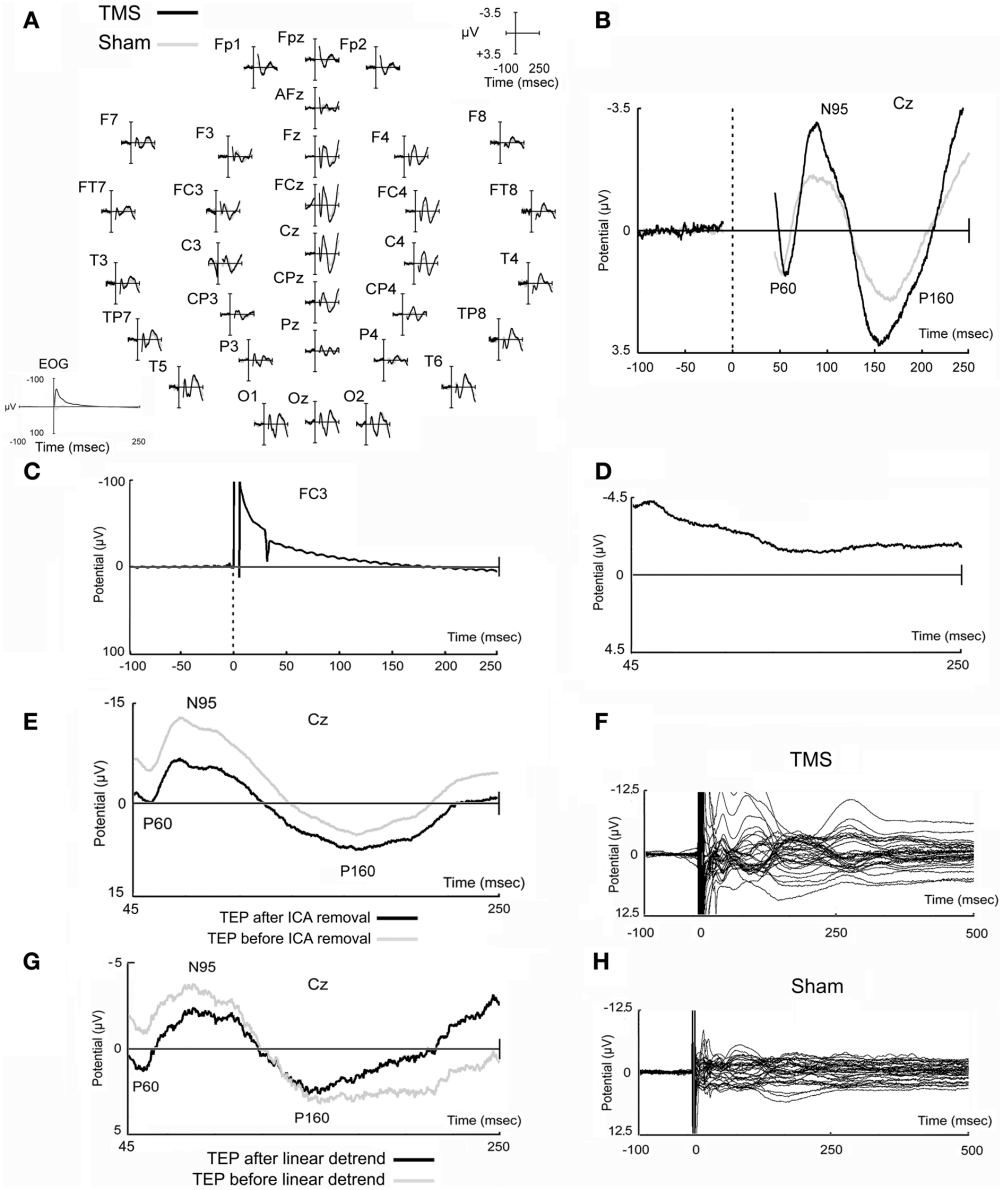
**Real and sham TMS-evoked potentials**. Evoked potentials obtained in real and sham TMS conditions as recorded in all electrodes **(A)** and in the representative Cz electrode **(B)**. In EOG data, TMS artifact is fully reported and appears longer likely due to filtering settings **(A)**. Potential that has been obtained in a prototypical electrode after the stimulation of the watermelon is also shown **(C)**, as well as a prototypical independent component, evidently related to TMS artifacts, that has been removed from data **(D)**. A TMS-evoked potential of a prototypical subject before and after ICA removal is also represented **(E)**. Finally, the butterfly plots of the grand-averaged TMS and sham raw data (before ICA decomposition and removal, and before linear detrend) are shown **(F,H)** in order to highlight that data tend to the baseline after the last components that appeared at about 250–300 ms after the delivery of the magnetic stimuli. An example of data before and after linear detrend is also shown **(G)**.

EEG traces were acquired by using a commercially-available system (MIZAR-SIRIUS system, acquisition software Galileo NT, EBNeuro, Italy). Specifically, an amplifier compatible with magnetic resonance acquisition (BASIS BE, EBNeuro, Italy) was used. Subjects wore an EEG elastic cap with 32 flat electrodes (Bionen sas, Italy). Electrode positions corresponded to classical positions and are reported in Figure 1A. More specifically, the following sensor positions were placed on the scalp: Fp1, Fp2, Fpz, AFz, F7, F3, Fz, F4, F8, FT7, FC3, FCz, FC4, FT8, T3, C3, Cz, C4, T4, TP7, CP3, CPz, CP4, TP8, T5, P3, Pz, P4, T6, O1, Oz, O2. The reference electrode was positioned between the AFz and Fz electrode, while the ground electrode was placed between the Pz and Oz electrode. EEG impedances were maintained under 10 KΩ. An electro-oculogram (EOG) was also acquired to allow accurate selection and rejection of noisy epochs. For this purpose, surface Ag/AgCl electrodes were placed above (near the external canthi) and below the right eye. Specific hardware and software settings were used to limit the impact of the TMS artifact on EEG traces. In particular, the sampling rate was set at 4096 Hz, with an analog band-pass filtering of 0.01–1843.2 Hz. The acquisition range was adjusted at ± 65500 μV to limit amplifier saturation. Moreover, electrode wires were carefully placed to limit the influence of TMS pulses on the EEG signal (Sekiguchi et al., 2011). Raw data were subsequently marked (real or sham TMS trials) and digitally filtered (band pass infinite impulse response filter 0.01–1000 Hz) using Neuroscan software (Compumedics Neuroscan Inc., El Paso, USA). EOG data were further elaborated by using a similar low pass filter at 50 Hz. Data were then segmented in epochs, considering a time window between −100 and 500 ms with respect to the delivery of TMS (0 ms) and corrected for baseline (from −100 to −10 ms before the delivery of the TMS pulse). Epochs were subsequently subdivided according to the considered condition (real or sham TMS) and visually inspected to discard those that presented excessive noise. In this sense, epochs that presented, for example, blink artifacts or that showed the presence of a drift that did not allow a reliable alignment of the trace (even if a baseline correction was performed) were not considered for further analyses. An average of 137.3 (*SD* = 21.3) epochs per subject was accepted for the real TMS condition, while 144.7 (*SD* = 25.8) epochs per subject were considered for the sham TMS condition. These epochs, grouped by conditions (real and sham TMS), were further analyzed with EEGLAB (Delorme and Makeig, 2004). An independent component analysis (Jung et al., 2000) was conducted to reduce the TMS-induced electric artifact and increase the quality of recorded data (e.g., Hamidi et al., 2011). Because of the large TMS artifact, which would highly impair the decomposition in independent components, ICA was performed considering a time range between 45 and 250 ms after delivery of the magnetic pulse. This approach allowed excluding most of the TMS artifacts that appeared immediately after TMS delivery (fast rising/decaying peak of signal, recharging artifacts, etc.) and, as much as possible, the residual part of the TMS artifact, and specifically the slow recovery of the signal after the delivery of the pulses (see Figures 2D,E).

It has been decided to reduce the considered time window of analysis until 250 ms after the delivery of the magnetic pulse in order to have a more direct comparison with previous works (Zanon et al., 2010; Busan et al., 2012). In fact, in these previous studies (Zanon et al., 2010; Busan et al., 2012) the more interesting results were mainly comprised in similar time ranges. A longer time window (see above) was initially considered only to allow a more reliable epochs selection for subsequent analyses even if, generally, it was noted the presence of further components (see Figures 2F,H), preferably stronger in the real TMS condition. For example, the component appearing after about 250–300 ms from TMS delivery, could be ascribed as an evidence arising from the stimulation of motor networks (e.g., Massimini et al., 2005; Ferreri et al., 2011), even if the possibility remains that it also arises from acoustic stimulation (e.g., Nikouline et al., 1999).

A further step was implemented for eliminating as much as possible the remaining TMS artifact: after averaging the epochs and obtaining real and sham TMS-evoked potentials, a “linear detrend” function was applied when needed (e.g., Van Der Werf and Paus, 2006). Averaged real and sham TEPs were re-referenced to a common average reference based on all the recorded 32 electrodes. Finally, grand-averaged TEPs were visually inspected by means of a butterfly-plot representation: this allowed to highlight the more evident time windows of analysis in a time range comprised between 45 and 250 ms (see below the sLORETA analysis section). In every time window, the relative local maxima peak of amplitude (and its latency) was highlighted in a series of representative electrodes: F3, Fz, F4, FC3, FCz, FC4, C3, Cz, C4, CP3, CPz, CP4, P3, Pz, and P4. Amplitudes (in μV; with respect to the zero value), as well as latencies (in ms; with respect to the delivery of TMS), were averaged among electrodes and considered as dependent variables. Data were successively analyzed by means of repeated measures ANOVA by considering main effects and interactions between time windows and stimulation condition (TMS vs. sham) for every dependent variable. *Post-hoc* analyses were conducted by using *T*-test. Significance was set at *p* < 0.05. Data were checked for their normality by means of the Shapiro–Wilk test and corrections were used in order to manage data that did not respect the assumption of sphericity and in order to correct for multiple comparisons.

Characterization of the TMS-induced artifact on the EEG signal was also performed on a watermelon, using the highest TMS intensity used in the present experiments and following the suggestions of Veniero et al. (2009). In this case, all EEG settings were similar to those used for real and sham TMS experiments.

### sLORETA analysis

Although scalp topography of electric potentials can provide some information about underlying neuronal dynamics, the distribution of EEG signal sources better allows investigation about the spatial localization of activated areas (Michel et al., 2004; He et al., 2011). Therefore, EEG source imaging was applied to both real and sham TMS evoked-potentials and a statistical comparison was performed between these conditions.

Standardized low resolution brain electromagnetic tomography (sLORETA; Pascual-Marqui, 2002) implemented in sLORETA-Key software (http://www.uzh.ch/keyinst/loreta.htm) was used to reconstruct the cortical three-dimensional distribution of the neuronal activity underlying real and sham TEPs. The sLORETA algorithm is a standardized discrete, three-dimensional distributed, linear, minimum norm inverse solution (Pascual-Marqui, 2002). Computations were realized in a realistic head model (Fuchs et al., 2002) based on the MNI152 template (Mazziotta et al., 2001), with three-dimensional space solution restricted to cortical gray matter, as determined by using the probabilistic Talairach atlas (Lancaster et al., 2000). Therefore, the intracerebral volume considered for the analysis comprised 6239 voxels at a 5-mm spatial resolution. Finally, electrode positions were superimposed on the MNI152 scalp (Oostenveld and Praamstra, 2001; Jurcak et al., 2007) and localization error was reduced by applying a regularization factor in all source reconstructions. Specifically, the mean signal-to-noise ratio in ERPs for each subject and condition was computed using the method of the 20th percentile, in a time window between 45 and 250 ms after stimulus onset. Anatomical labels and Brodmann areas were reported in the MNI space, with the possibility to correct to the Talairach space (Brett et al., 2002).

A voxel-by-voxel within-subject comparison of EEG sources was performed, and significant differences in real and sham TMS conditions were assessed with non-parametric statistical analysis based on a permutation test (Statistical non-Parametric Mapping: SnPM; Nichols and Holmes, 2002), implemented in sLORETA-Key software. Both *t*-statistic and log of F-ratio were computed, to obtain a more comprehensive evaluation of the possible activations elicited by the present protocol.

Furthermore, statistical analyses were conducted comparing real and sham TMS signals in each time-frame (time-frame by time-frame analysis) and the mean source signal in selected time intervals of interest (mean signal analysis). In both cases, analyses were restricted to time windows ranging from 45 to 213 ms after stimulation, chosen after visual inspection of the grand-average butterfly plot of real TMS and sham TEPs and in order to limit possible biases related to the application of the linear detrend function, especially when considering the end of the original time window of interest (i.e., 250 ms after TMS delivery). Thus, this window was further subdivided in 3 windows of interest: from 45 to 70 ms, from 70 to 130 ms, and from 130 to 213 ms. Significance was set at *p* < 0.05; this threshold was conservatively corrected with respect to the number of time windows that were considered in each analysis. Importantly, SnPM in sLORETA automatically allowed for correction of multiple comparisons in each computed analysis even with respect to all examined voxels and time samples. When considering time-frame by time-frame analyses, in order to assure a greater confidence in results obtained from the main statistical analyses, we also reported the number of time-frames that resulted in a consecutive activation, at a trend level (*p* < 0.1; see Tables 1, 2), of every maximal peak of activation obtained from main analyses. This has been done considering that reliable and meaningful brain activations should occur in microstates.

**Table 1:** **Results from time-frame by time-frame sLORETA analysis (t-statistic)**.

**Time of activation (ms)**	**Maximal peak of activation (MPA)**			**Other significant voxels (BA)**	**Number of activated voxels (mean and *SD*)**	**Number of MPA time-frames (~ms) at *p* < 0.1**
	***X, Y, Z* (MNI coordinates)**	***BA***	**Anatomical landmark**			
59–60	−65, −15, −5	21	Left middle temporal gyrus	/	3 (1.4)	8 (~2)
132–133	45, −20, 40	4	Right pre-central	/	1 (0)	22 (~5.5)
	40, −20, 40		gyrus			19 (~5)
134–137	45, −20, 40	4, 9,	Right pre-central	2 R, 3 R, 6 R, 8 R, 10	14.8 (13.8)	22 (~5.5)
	40, 35, 35	46	gyrus,	R, 24 R, 32 R, 40 R,		18 (~4.5)
	45, 40, 30		Right superior	46 R		22 (~5.5)
	35, 30, 35		frontal gyrus,			24 (~6)
	35, 25, 35		Right middle			26 (~6.5)
	45, 30, 25		frontal gyrus,			21 (~5)
	35, 20, 35		Right inferior			25 (~6)
	35, 5, 30		frontal gyrus			19 (~5)
	30, 35, 30					19 (~5)
138–139	35, 40, 25	10	Right middle	9 R	4 (1)	18 (~4.5)
	40, 45, 30		frontal gyrus			17 (~4.5)
140	30, 20, 15	13	Right insula	46 R	4 (–)	9 (~2)

*Time of activation and location of maximal peaks that were significant with analysis made on discrete time windows of interest. The remaining significant voxels are also reported by indicating BA. BA, Brodmann Area; L, left; R, right*.

**Table 2:** **Results from time-frame by time-frame sLORETA analysis (log of F-ratio)**.

**Time of activation (ms)**	**Maximal peak of activation (MPA)**			**Other significant voxels (BA)**	**Number of activated voxels (mean and *SD*)**	**Number of MPA time-frames (~ms) at *p* < 0.1**
	***X, Y, Z* (MNI coordinates)**	***BA***	**Anatomical landmark**			
61–65	−45, 10, 55	6, 9	Left middle frontal gyrus	8 L	10.1 (8.9)	29 (**~**7)
	−45, 30, 40					27 (**~**7)
88–89	−35, 50, 30	10	Left superior frontal gyrus	46 L	2.3 (0.58)	40 (**~**10)
	−35, 55, 20					34 (**~**8)
90	−35, 55, 20	10	Left superior frontal gyrus	46 L	3 (3.5)	34 (**~**8.5)
	−35, 50, 30					40 (**~**10)
199	−45, 10, 55	6	Left middle frontal gyrus	1 L, 2 L, 3 L, 8 L, 40 L	20 (–)	21 (**~**5)

*Time of activation and location of maximal peaks that were significant with analysis made on discrete time windows of interest. The remaining significant voxels are also reported by indicating BA. BA, Brodmann Area; L, left; R, right*.

### Anatomical localization

In order to verify the cortical areas beneath the selected stimulation site, TMS coil position on the scalp of a prototypical subject was marked with a vitamin E pill. Immediately after, magnetic resonance imaging (MRI) anatomical acquisition was performed (T2-weighted, slide thickness 4 mm, TR = 3833.13 ms, TE = 100 ms, fov 230 × 230, acquisition matrix 256 × 256 pixels, pixel spacing 0.4 × 0.4).

## Results

### Real and sham TMS-evoked potentials

Figure 2 reports typical TEPs from all electrodes (Figure 2A) and from Cz electrode (Figure 2B), to show, in more detail, the main peaks of activity we observed. On this representative electrode, after real TMS, a first positive component (mean amplitude 2.29 μV; SD 2.34), named P60, appeared at a mean latency of 57.1 ms (SD 3.9), followed by a negative component (mean amplitude −4.42 μV; SD 3.43), named N95, with a mean peak latency of 95.2 ms (SD 23.6), and by a second positive component (P160) at 158.7 ms (SD 20.5), with a mean amplitude of 4.87 μV (SD 2.75).

Sham evoked potentials showed similar deflections, but with reduced amplitudes compared to real TMS (Figures 2A,B). A positive component was observed at 54.6 ms (SD 5.7), with a mean amplitude of 1.61 μV (SD 1.10); a negative component appeared at a mean latency of 92.0 ms (SD 16.8) with a mean amplitude of −2.23 μV (SD 0.81); the third, positive component was detected at 169.3 ms (SD 17.3) with a mean amplitude of 2.75 μV (SD 1.28). The strict similarity of waves can be ascribed to acoustic contamination (Nikouline et al., 1999).

Mean real TMS amplitudes obtained for every time window of interest on a series of representative electrodes (see Materials and Methods section) were 1.18 μV (SD 0.92), −2.18 μV (SD 1.41), and 2.17 μV (SD 1.24), respectively. On the other hand, mean sham amplitudes resulted 0.91 μV (SD 0.72), −1.11 μV (SD 0.42), and 1.31 μV (SD 0.55), respectively. Statistical analyses showed that amplitudes were different with respect to the different time windows considered [*F*_(1.149, 12.640)_ = 46.252, *p* < 0.0009] and also an interaction between stimulation condition (TMS vs. sham) and time window of interest was present [*F*_(2, 22)_ = 7.183, *p* = 0.004]. *Post-hoc* analyses showed that real TMS amplitudes were significantly higher with respect to sham amplitudes when considering the second and the third time window of interest [*t*_(11)_ = 3.26, *p* = 0.008; *t*_(11)_ = 2.92, *p* = 0.014, respectively].

**Figure 3:**
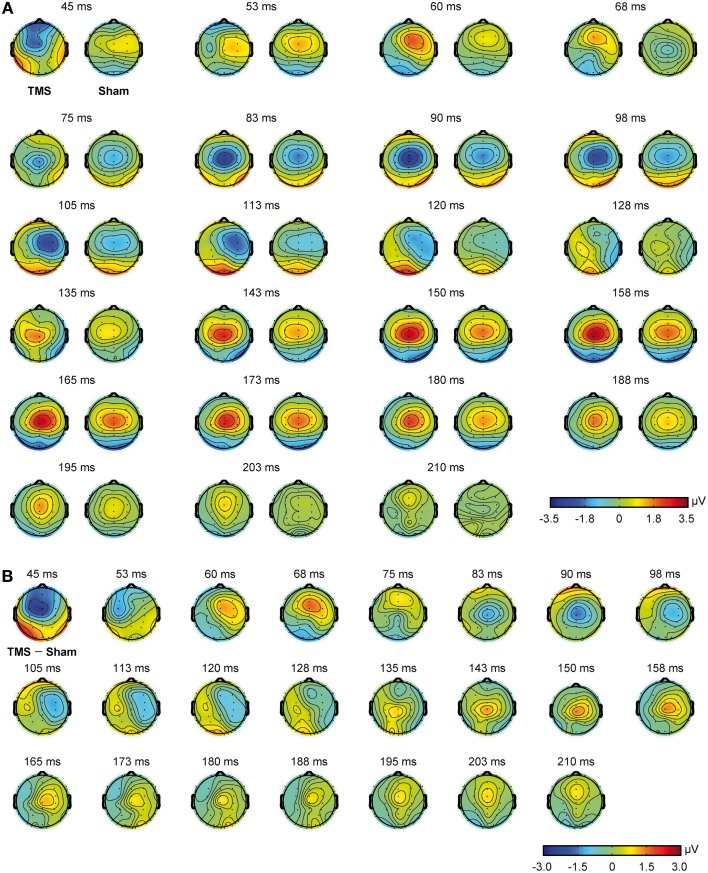
**Scalp topographies obtained from real TMS and sham conditions**. Real TMS scalp topographies are shown on the left and sham topographies on the right **(A)**. Scalp topographies obtained from the direct comparison of real TMS and sham conditions are also shown **(B)**.

Mean real TMS latencies on a series of representative electrodes, for every time window of interest (see Materials and Methods section), were 60.6 ms (*SD* = 4.3), 101.6 ms (*SD* = 13.4), and 164.5 ms (*SD* = 14.2), respectively. On the same line, mean sham latencies resulted 55.5 ms (*SD* = 4.5), 99.8 ms (*SD* = 13.2), and 166.2 ms (*SD* = 12.5), for every time window of interest. Statistical analyses showed that latencies were different with respect to the different time windows of interest [*F*_(2, 22)_ = 635.013, *p* < 0.0009], but the interaction between stimulation condition and time window of interest resulted only in a trend toward significance [*F*_(2, 22)_ = 2.865, *p* = 0.078]. Scalp topographies, with respect to the different conditions, are also shown in Figure 3.

Stimulation of the watermelon, carried out according to Veniero et al. (2009), showed that a slower artifact was evident, after the initial and greater fast-rising and fast-decaying TMS-related artifact, which lasted several ms after TMS administration. Unfortunately, we found it difficult to properly reduce the impedances, which were generally worse than those obtained from the subject's scalp. Therefore, whereas proper conclusions cannot be achieved, we believe that impedance might influence the size and the duration of the TMS-related artifacts (for a discussion see Julkunen et al., 2008; Veniero et al., 2009). Finally, a further artifact was evident that was related to the TMS recharge after delivery of the stimulus. A prototypical characterization of the artifacts related to the delivery of TMS on watermelon is showed in Figure 2C.

### sLORETA: time-frame by time-frame analysis

Non-parametric time-frame by time-frame statistical tests showed significantly different cortical activations between real and sham TMS conditions, in a time window between 45 and 213 ms.

**Figure 4:**
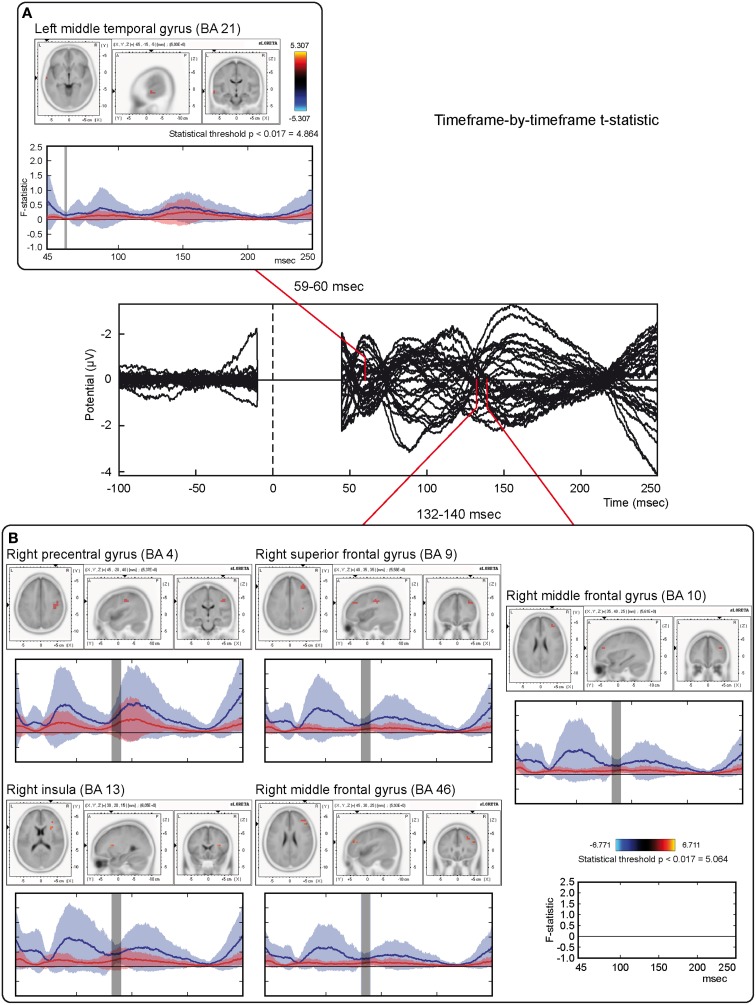
**Time-frame by time-frame sLORETA results (1)**. Principal sLORETA results obtained when considering a time-frame by time-frame analysis performed with *t*-statistic and comparing real TMS minus sham conditions. Images are plotted with respect to the time windows identified in a butterfly plot of the evoked potentials obtained from the real TMS condition. **(A)** Results obtained in the 59–60 ms time window. Activation with the maximal peak in the left middle temporal gyrus (BA 21) is shown. **(B)** Results obtained in the 132–140 ms time window. Activations with the maximal peaks in the right sensorimotor and frontal regions (BA 4, 9, 10, 13, and 46) are shown. The time course of the intensity of the signal in the source space is also shown. Specifically, real TMS signals for a specific peak (corresponding to a specific voxel) are indicated by a blue line (standard deviations are indicated by shadows of the same color). Sham signals for the same peak (and voxel) are indicated by a red line (standard deviations are indicated by shadows of the same color). The corresponding significant time-frames are indicated by gray shadows. It is very important to note that the intensity of the signal in the source space has, here, the form of an F-statistic, since sLORETA perform the standardized estimate of the cortical current density, expressed as a statistical value (F-distribution value; Pascual-Marqui, 2002).

Tests based on *t*-statistic (Table 1 and Figure 4) revealed significant neuronal activity induced by real TMS (minus sham TMS) at about 60 ms after stimulus delivery and in an interval roughly between 132 and 140 ms. The left temporal cortex (middle temporal gyrus; BA 21) was more active in real TMS (minus sham TMS) at early time points, whereas at later time intervals differences were observed in the sensorimotor regions of the right hemisphere, contralateral to the side of stimulation. Voxels with significant differences were observed in the right motor regions (pre-central gyrus; BA 4 and 6) as well as in somatosensory areas (right post-central gyrus; BA 2 and 3). In addition, significant voxels were detected in the right superior, middle, and inferior frontal gyri (BA 9, 6, 8, 10 and 46), in the right inferior parietal lobule (BA 40), and in the right cingulate gyrus (BA 24 and 32). Finally, toward the end of the time window, greater activation for real TMS (minus sham TMS) was observed in the right insula (BA 13).

**Figure 5:**
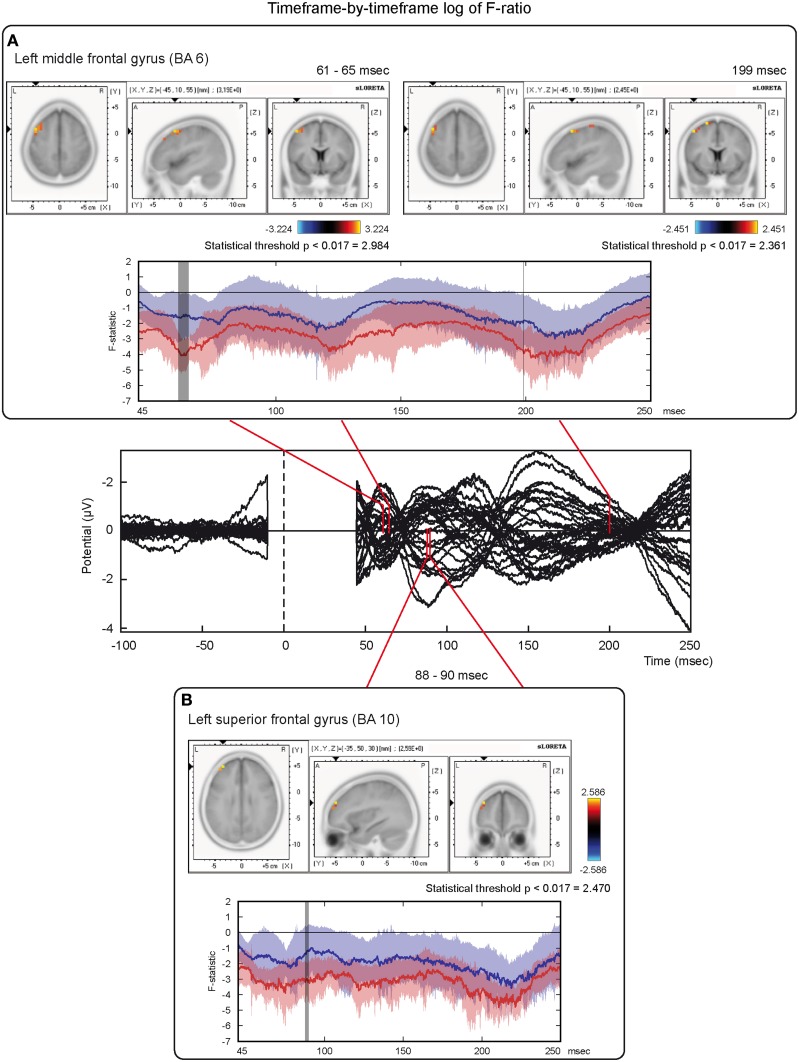
**Time-frame by time-frame sLORETA results (2)**. Principal sLORETA results obtained when considering a time-frame by time-frame analysis performed with log of F-ratio and comparing real TMS minus sham conditions. Images are plotted with respect to the time windows identified in a butterfly plot of the evoked potentials obtained from the real TMS condition. **(A)** Results obtained in the 61–65 ms time window and results obtained 199 ms after the delivery of TMS. Activations with the maximal peak in the left middle frontal gyrus (BA 6) are shown. **(B)** Results obtained in the 88–90 ms time window. Activation with the maximal peak of activation in the left superior frontal gyrus (BA 10) is shown. The time course of the intensity of the signal in the source space is also shown. Real TMS signals for a specific peak (corresponding to a specific voxel) are indicated by a blue line (standard deviations are indicated by shadows of the same color). Sham signals for the same peak (and voxel) are indicated by a red line (standard deviations are indicated by shadows of the same color). The corresponding significant time-frames are indicated by gray shadows. It is very important to note that the intensity of the signal in the source space has, here, the form of an F-statistic, since sLORETA perform the standardized estimate of the cortical current density, expressed as a statistical value (F-distribution value; Pascual-Marqui, 2002). It has negative values because they are the logarithmic transformation of source activation estimates.

On the other hand, analyses based on log of F-ratio (Table 2 and Figure 5) revealed that real TMS (minus sham TMS) induced greater activation (significant voxels) mainly in the premotor (BA 6) and frontal regions (BA 9 and 8) of the stimulated left hemisphere (left middle frontal gyrus, left superior frontal gyrus, and left pre-central gyrus), in a time range roughly between 61 and 65 ms after stimulation. Furthermore, left frontal regions and sensorimotor networks showed significantly higher activations in a time interval roughly between 88 and 90 ms, and around 200 ms after real TMS (minus sham TMS). Significant voxels were observed in the left superior and middle frontal gyri (BA 10 and 46), while left middle and superior frontal gyri, pre- and post-central gyri, and the left inferior parietal lobule (BA 6, 8, 1, 2, 3, and 40) were more active in the subsequent time points. Tables 1, 2 report also the number of consecutive time-frames that resulted toward a significant activation, at a trend level (*p* < 0.1), of every maximal peak of activity highlighted from main analyses.

### Mean neural activity in specific time windows of interest

**Figure 6:**
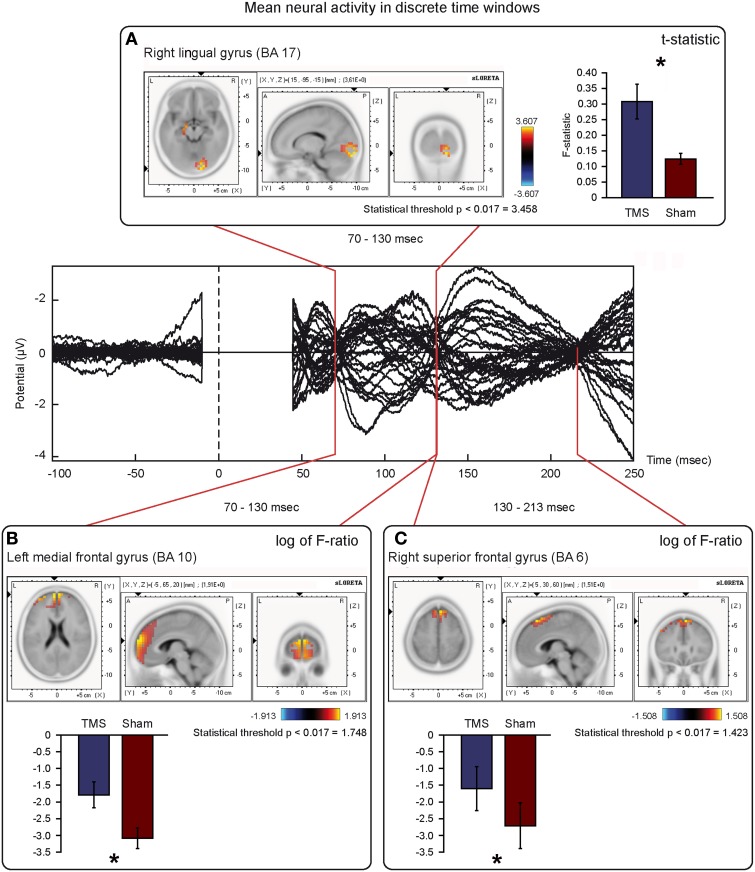
**Mean neural activity in discrete time windows of interest**. Principal sLORETA results obtained when considering the mean of neural activity in discrete time windows and analysis performed with *t*-statistic **(A)** and log of F-ratio **(B,C)**. Statistics have been performed comparing the real TMS minus the sham conditions. Images are plotted with respect to the time windows identified in a butterfly plot of the evoked potentials obtained from the real TMS condition. **(A)** Mean results obtained in the 70–130 ms time window with *t*-statistic. Activation with the maximal peak in the right lingual gyrus (BA 17) is shown. **(B)** Mean results obtained in the 70–130 ms time window with log of F-ratio. Activation with the maximal peak in the left medial frontal gyrus (BA 10) is shown. **(C)** Mean results obtained in the 130–213 ms time window with log of F-ratio. Activation with the maximal peak in the right superior frontal gyrus (BA 6) is shown. The mean intensity of the signal in the source space in the time window of interest is also shown. Averaged real TMS signal for a specific peak (corresponding to a specific voxel) is indicated by a blue bar (standard deviations are also indicated). Averaged sham signal for the same peak (and voxel) is indicated by a red bar (standard deviations are also indicated). It is very important to note that the intensity of the signal in the source space has, here, the form of an F-statistic, since sLORETA perform the standardized estimate of the cortical current density, expressed as a statistical value (F-distribution value; Pascual-Marqui, 2002). It has negative values in panels **(B)** and **(C)** because they represent the logarithmic transformation of mean source activation estimates. Asterisks indicate a significant difference between conditions.

Mean activations in the three time windows of interest induced by real and sham TMS were compared with paired-sample non-parametric tests, using both *t*-statistic and log of F-ratio tests. Tables 3, 4 summarize the main results, while Figure 6 shows the main data over models of structural MRI.

**Table 3:** **Results from discrete time windows sLORETA analysis (mean neural activity, *t*-statistic)**.

**Time of activation (ms)**	**Maximal peak of activation**			**Other significant voxels (BA)**	**Number of activated voxels**
	***X, Y, Z* (MNI coordinates)**	***BA***	**Anatomical landmark**		
45–70	N.S.	N.S.	N.S.	N.S.	N.S.
70–130	15, −95, 15	17	Right lingual gyrus	6 L, 9 L, 18 R, 19 R, 23 R, 28 L, 30 R, 34 L, 35 L, 36 L,	123
130–213	N.S.	N.S.	N.S.	N.S.	N.S.

*Time of activation and location of maximal peaks that were significant are reported. The remaining significant voxels are also reported in BA. BA, Brodmann Area; L, left; R, right; N.S., Not Significant, results did not reach threshold for significance*.

**Table 4:** **Results from discrete time windows sLORETA analysis (mean neural activity, log of F-ratio)**.

**Time of activation (ms)**	**Maximal peak of activation**			**Other significant voxels (BA)**	**Number of activated voxels**
	***X, Y, Z* (MNI coordinates)**	***BA***	**Anatomical landmark**		
45–70	N.S.	N.S.	N.S.	N.S.	N.S.
70–130	−5, 65, 20	10	Left medial frontal gyrus	6 L/R, 8 L/R, 9 L/R, 10 R, 11 L/R, 32 L/R, 46 L	360
130–213	5, 30, 60	6	Right superior frontal gyrus	6 L, 8 L/R, 9 L	69

*Time of activation and location of maximal peaks that were significant are reported. The remaining significant voxels are also reported in BA. BA, Brodmann Area; L, left; R, right; N.S., Not Significant, results did not reach threshold for significance*.

T-statistic revealed significant differences only in the second time window of interest between 70 and 130 ms after real TMS (minus sham TMS) delivery. Analyses between 45 and 70 ms and between 130 and 213 ms after stimulus delivery did not reach the significance threshold. Significant voxels were mainly evident in the right hemisphere: the lingual gyrus, the middle and inferior occipital gyri, the fusiform gyrus, and the cuneus (BA 17, 18, 19, 23, 30). Moreover, in the same window, real TMS (minus sham TMS) induced significantly different activity in the left uncus, left parahippocampal gyrus (BA 28, 34, 35, 36), left pre-central gyrus, and in left middle and inferior frontal gyri (BA 6 and 9).

Statistical analyses based on log of F-ratio demonstrated significant differences between real TMS and sham conditions in two time windows (70–130 ms and 130–213 ms after stimulus delivery), while activity in the 45–70 ms time window did not reach the threshold for significance. After 70–130 ms, significant voxels were evident in the left hemisphere and also in the right hemisphere. Significant voxels in the real TMS condition (minus sham TMS) were observed bilaterally in the medial frontal gyrus, superior and middle frontal gyri, orbital gyrus and anterior cingulate cortex, and in the left cingulate, rectal, and inferior frontal gyri. These patterns of activity corresponded to BA 10, 6, 8, 9, 11, 32, and 46. When considering the time range between 130 and 213 ms, significant voxels were bilaterally evident in the superior frontal gyrus and medial frontal gyrus, as well as in the left middle frontal gyrus (BA 6, 8, 9).

### Anatomical localization of the stimulation site

Visual classification of brain gyri and sulci of a prototypical subject was performed on the basis of Duvernoy (1999) on an MRI scan. A vitamin E pill was used as fiducial and its position on the scalp allowed confirmation that the stimulation site successfully comprised the dorsal premotor cortex, as shown in the right panel of Figure 1. In this sense, we should also consider that the projection of particular EEG points on cortical surface could be estimated with an average standard deviation of 8 mm in a previous work by Okamoto et al. (2004).

## Discussion

### Summary of results and general interpretation

Our findings might depict the spatial and temporal long-range neural activity evoked by premotor cortex stimulation. In fact, PMd stimulation evoked significant activations in discrete and different time windows of interest, comprised in a larger window of analysis ranging from 45 ms to about 200 ms after stimulus delivery, which can be interpreted as evidence for the connectivity between the PMd and several other brain regions, mainly bilaterally within the frontal lobes, and with the posterior, occipital pole. Specifically, the main pattern of activity suggests stimulus propagation toward contralateral brain regions, mainly in the sensorimotor and frontal areas, within discrete time windows, together with the activation of more posterior, principally visual, brain areas. Moreover, a pattern of mainly ipsilateral activations was also found in sensorimotor and/or frontal structures. Thus, significant voxels were found both in the same hemisphere as well as contralaterally to the stimulation site. In the first case, both cortico-cortical connections and/or subcortical pathways possibly contributed to the pattern of activations (e.g., Zanon et al., 2010; Busan et al., 2012), whereas in the latter case the flow of TMS-induced activation was likely carried by transcallosal connections (Marconi et al., 2003).

Propagation of activity from the left premotor cortex has already been investigated using TMS/EEG (e.g., Casarotto et al., 2010; Korhonen et al., 2011). However, to the best of our knowledge, only discrete and early activations were considered (e.g., Casarotto et al., 2010), or the study focused on the methodological aspects of TMS/EEG (e.g., Korhonen et al., 2011). On the other hand, when TMS and/or TMS/EEG have been used to investigate the premotor/motor network, the propagation of activity from the primary motor cortex, rather than from PMd, was the main focus (e.g., Komssi et al., 2004; Esser et al., 2006).

The definition of connections among different areas is crucial not only to understand the functional organization of neural systems, but it can also be helpful to investigate disease mechanisms, as in the case of the spread of epileptic discharges to gather new information for planning surgical intervention (e.g., Engel et al., 2003).

Functional connections could be present between brain regions that are both directly linked by axonal fibers and/or through indirect pathways. Especially when a large interregional distance is involved, the latter may explain the degree of variance in functional connectivity that cannot be fully described by structural connectivity. Indeed, findings here reported could be more properly related with long-range neural activity. Moreover, this suggests that connectivity could be variable in time (e.g., Bestmann et al., 2008; Moisa et al., 2012) and different cortical regions could work together forming functional connections according to the specific tasks they have to process. In this view, the PMd may form a functional network with frontal, parietal, and/or occipital cortical regions, varying with specific type of processes, like for example the planning of internally-paced rather than visually-guided sequence of movements (e.g., Bestmann et al., 2008; Moisa et al., 2012).

In the present study, several components characterized the electrical potentials evoked by PMd stimulation, namely the P60, the N95 and the P160, as described in the Results section. They are similar to those previously described in other TMS/EEG experiments, where TMS was applied to different regions of cortex (Paus et al., 2001; Komssi et al., 2002, 2004; Bonato et al., 2006; Lioumis et al., 2009; Zanon et al., 2010; Ferreri et al., 2011; Busan et al., 2012). Consistent with previous studies, EEG deviations from pre-stimulus baseline could be induced by activation of local and remote cortical neurons and/or synchronization of ongoing activity (e.g., Paus et al., 2001; Groppa et al., 2013). Further components should not be excluded, and in particular in the time windows not considered in the present analysis, for example those between 0 and 45 ms, which were removed because the recorded potentials were still corrupted by the electric artifact induced by the strong magnetic pulse.

### Propagation of activity toward contralateral regions

The novelty of the present investigation relies on the long-range of the spatial and temporal profile of the activity propagation that was evoked when stimulating the dorsal premotor cortex in the left hemisphere. Furthermore, it provides new evidence to the presence of homo- and heterotopic projections that underpin the exchange of information inside the hemispheres and between them.

Stimulation of the left PMd showed, at about 130 ms from stimulus delivery, long-range contralateral activations mainly in the frontal and sensorimotor cortices (see Figure 4B). The present findings partially confirm what was already observed when investigating PMd connectivity with different techniques. For example, when TMS was applied on PMd during functional MRI (fMRI), a significant BOLD signal increase was observed in regions such as the contralateral PMd, bilateral premotor ventral regions, somatosensory cortex or the supplementary motor area, as well as in subcortical regions, such as the cerebellum and/or the caudate nucleus (e.g., Bestmann et al., 2005).

Contralateral propagation of activity during TMS/EEG co-registration was observed by Komssi et al. (2002) by stimulation of the left sensorimotor cortex, detecting activity in contralateral homologue brain regions, especially when considering the first 30 ms after the delivery of TMS and ipsilateral activations mainly in the sensorimotor structures and parietal lobe. In this regard, Ilmoniemi et al. (1997) showed activation of the contralateral homologous cortex about 20 ms after the delivery of the magnetic stimulus over central regions. Interestingly, observations similar to those reported in the present study (e.g., the transmission of the signal toward contralateral sensorimotor and frontal regions in discrete time windows of interest), were also obtained by Massimini et al. (2005) using a similar eyes-closed approach. For instance, they found contralateral activation of frontal networks when stimulating a dorsal premotor region in the right hemisphere, also in comparable time windows of interest. They could speculatively represent late and reverberant communications between frontal and sensorimotor regions of the two hemispheres, which are commonly active in the performing of specific tasks, like for example motor/cognitive tasks (e.g., Bestmann et al., 2008), and here engaged in the considered time window of interest by the initial and particular mental state of the brain (i.e., resting with closed eyes). Casarotto et al. (2010), in a study designed to evaluate the sensitivity and the repeatability of induced TEPs, performed a TMS/EEG investigation stimulating a very medial left dorsal premotor/supplementary motor region. The analysis of the neural generators (until 80 ms after stimulus delivery) showed that activations mainly propagated around the site of stimulation and toward more frontal regions with respect to contralateral and/or posterior regions. Finally, Iwahashi et al. (2008) observed the propagation of activity in a very early time window (about 20 ms after the stimulation of motor and parietal regions in both hemispheres) toward various anterior and posterior regions of the contralateral hemisphere, especially when stimulating motor regions.

Although the activations found in the sensorimotor cortex could be related to a somatosensory/peripheral effect evoked by TMS (see Paus et al., 2001, for a discussion; Ruff et al., 2006), it was suggested that TEPs were not significantly contaminated by somatosensory/peripheral effects after left primary motor cortex stimulation (e.g., Paus et al., 2001). In any case, the possibility of a somatosensory/peripheral stimulation related with TMS should be always considered (e.g., Ruff et al., 2006).

### Propagation of activity toward frontal regions

Our findings suggest a pattern of activations that could appear, at a first view, mainly evident in the left hemisphere (Figure 5) in sensorimotor and/or frontal structures. The suggested connectivity among PMd and regions in the frontal and/or pre-frontal cortex is in agreement with the hypothesis of a rostro-caudal axis in the organization of the premotor cortex. According to this theory, rostral subdivisions are mainly involved in abstract and higher-order processes, while the caudal ones are mainly involved in low-order and motor-related processes (e.g., Gangitano et al., 2008; Goulas et al., 2012; Nee and Brown, 2012). More specifically, the rostral portion of the dorsal premotor cortex could be also seen as pre-PMd, while its caudal portion could be indicated as the proper PMd. This might reflect a parallelism between these areas and the pre-supplementary motor area (pre-SMA)/SMA complex. Thus, the pre-PMd seems to be preferentially involved in the cognitive aspects of brain functioning and more interconnected with other frontal regions, while the proper PMd seems to be more tightly connected with the motor aspects of behavior, such as the spatial and temporal characteristics of muscle activation (e.g., Picard and Strick, 2001; Matsumoto et al., 2003). As a consequence, activations related to higher-order processing, as could be the case of conditional visuo–motor associations, response selection, or motor imagery, should be mainly located in the pre-PMd (Grafton et al., 1998; Toni et al., 1999; Gerardin et al., 2000; Sakai et al., 2000), even if there is also substantial evidence suggesting an overlap between premotor regions that are important for both cognitive and motor tasks (Rottschy et al., 2012). Along these lines, the significant voxels we observed in regions in the frontal pole could be activated by the stimulation of rostral portions of PMd that are preferentially interconnected with more frontal brain regions (e.g., Barbas and Pandya, 1987; Lu et al., 1994). These regions could be involved in allocation of cognitive resources, selection of appropriate motor responses, and/or concurrent inhibition of unneeded ones (Chambers et al., 2007; Duque et al., 2012), speculatively supporting long-range timing of interactions, as in the present work.

The present pattern of results (e.g., the flowing of communication among different premotor and frontal regions) could also partially represent an interchange of information that could intervene between different eye fields that may be present in these regions of cortex (e.g., Luppino and Rizzolatti, 2000; see also Figures 5A, 6C). Finally, the dorsal premotor cortex could modulate the activity of the ventral premotor one: in fact, both areas could be interconnected (e.g., Goulas et al., 2012).

### Propagation of activity toward posterior brain regions

We observed propagation of activity from PMd to posterior, mainly visual, brain regions (Figure 6A), in agreement with the already mentioned observations of Massimini et al. (2005) and Iwahashi et al. (2008). In this sense, also findings reported by Chouinard et al. (2003) suggest the possibility that motor regions might influence the activity of posterior brain regions mainly related to vision. The antero-posterior communications within the brain have been viewed as reciprocal, allowing not only the serial but also the recursive coding of information (e.g., Paus et al., 1997; Battaglia-Mayer et al., 1998; Massimini et al., 2005). Accordingly, concurrent serial and parallel processing in the human brain is present during integration of inputs that are necessary to perform, for example, visually-guided behaviors (e.g., Busan et al., 2009b; Hinkley et al., 2011). With regards to the PMd, this cortical region is presumed to be part of a network comprising the premotor cortex and superior parietal lobule whose activities underpin the parallel and recursive exchange of information (e.g., Battaglia-Mayer et al., 2003; Naranjo et al., 2007; Moisa et al., 2012; Rottschy et al., 2012). This model could be further supported by the present finding that posterior brain regions are activated in a time window between 70 and 130 ms after the delivery of TMS on PMd. Furthermore, the neurobiological substrate of this exchange of information can be the dorsal visual stream (Colby et al., 1988; Tanne et al., 1995; Rizzolatti and Matelli, 2003; Milner and Goodale, 2006) or the occipito-frontal fascicle (Jellison et al., 2004; Makris et al., 2007; Forkel et al., 2012) in this particular time window of interest.

By using the same approach, Casali et al. (2010) showed that a maximum spread of activation in the ipsilateral frontal cortex occurred in a time interval roughly between 70 and 100 ms after the stimulation of left superior occipital regions. Moreover, intracranial recordings in monkeys (Schroeder et al., 1998; Lamme and Roelfsema, 2000) and humans (Gaillard et al., 2009) have also demonstrated that visual stimulation could result in a posterior to anterior propagation of neural activity that reached the ipsilateral frontal lobe at latencies mainly between 120 and 150 ms. It is likely that these networks and circuits are indeed more complex (e.g., Zanon et al., 2010; Busan et al., 2012), as fascicles are simply physical links.

Taken together, these data support the view that different regions in the brain have different patterns of connectivity with the bilateral ventral and dorsal extrastriate cortex, which might be the basis of the differential organization of action and/or cognition (Rottschy et al., 2012). In the present work, the prevailing stimulation of the rostral PMd would likely involve regions that are compatible with eye-related neural activity, as for example frontal eye fields (FEF; Paus et al., 1997; Ruff et al., 2009), supplementary eye fields (Luppino and Rizzolatti, 2000; Amiez and Petrides, 2009), premotor/cingulate eye fields with the elicitation of other eye-related activities induced by premotor stimulation (Amiez and Petrides, 2009). Interestingly, Hinkley et al. (2011) showed that early high-gamma activity over the FEF during the saccade preparation moved toward the visual cortex during saccade execution. Overall, these observations suggest the presence of a connection between these regions (Paus et al., 1997; Ruff et al., 2006, 2009).

In the present study, magnetic stimuli were delivered to subjects who had their eyes closed. Therefore, the spread of activity should be considered in the light of the state-dependent theory (e.g., Silvanto and Muggleton, 2008). The findings presented herein were obtained starting from a condition that could be considered as similar to a “default mode” neural condition (e.g., Raichle et al., 2001; Raichle and Snyder, 2007; Greicius et al., 2009). In recent years, a growing body of evidence has supported the hypothesis of the existence of long-range brain networks with interdependent activities, which likely underpins mental processes (Bressler and Menon, 2010). Because these networks are identified both at rest and/or during the execution of active tasks, each might represent a distinct and intrinsically organized functional network (Calhoun et al., 2008; Smith et al., 2009). In this light, we suggest that TMS should have activated remote brain areas that could be part of the same functional network. This hypothesis could explain the similar results (e.g., activation of posterior brain regions) we obtained after stimulation of other cortical regions that could be part of the same network (Busan et al., 2012).

### Limitations of the study

TMS/EEG is a challenging technique, mainly because the magnetic stimulus induces a strong electric artifact that corrupts the EEG traces for some ms after stimulus delivery (Virtanen et al., 1999; Rogasch and Fitzgerald, 2013). For this reason, in most of the previous TMS/EEG studies, the analysis of recorded electric potentials started several ms after the delivery of the TMS pulse (e.g., Komssi et al., 2002; Litvak et al., 2007; Zanon et al., 2010; Busan et al., 2012). Nevertheless, the TMS/EEG technique allows investigation of the neural changes that happen in brain regions that are related to the stimulated area by means of direct and/or indirect links (Ilmoniemi et al., 1997; Massimini et al., 2005; Daskalakis et al., 2012).

In addition, one should consider that TMS evokes not only responses directly related to the magnetic stimulation, but also potentials due to acoustic and somatic stimulations. In this study, two components were principally observed on central electrodes, named N95 and P160, and could be mainly related to acoustic stimulation related to TMS delivery (Nikouline et al., 1999). In fact, similar components were observed both after real and sham TMS (slightly reduced in amplitude in the sham condition). In any case, this observation suggests that sham TMS could be a reliable control for acoustic stimulation related to TMS delivery. On the other hand, a reliable control for the somatic sensation related to TMS is difficult to obtain. TMS evoked a tactile sensation on the scalp and excitation of sensory receptors might activate the somatosensory cortex, for example through the trigeminal pathway, thus confounding the results such as EEG source imaging. Nonetheless, the present significant activations for real TMS were not clearly compatible with the results reported in studies that investigated the neuronal sources of sensorimotor evoked potentials related to trigeminal activations (e.g., Ohla et al., 2010). Thus, even if we attempted to eliminate the majority of artifacts, also by using ICA, a gold-standard method (Jung et al., 2000), the possibility remains that some artifacts, even if reduced in strength, were still present in the data collected. Moreover, even if we eliminated the major part of the TMS-induced artifacts by cutting the first 45 ms of post-stimulus EEG traces, and other artifacts by visual inspection and ICA, slower TMS artifacts might still be present in the acquired data. Linear detrend (e.g., Van Der Werf and Paus, 2006; Zanon et al., 2010) was used to partially correct for this problem, but all these elements should be taken into account. Thus, the possibility remains that some of the present patterns of activations could be related to and/or influenced by a series of unspecific effects of the magnetic stimulation that could be difficult to individuate and control in a reliable manner. Also for these reasons, the present paradigm might not have revealed some activations that could be part of the network.

The present findings should be considered as based on long-range neural activity, both from a spatial and temporal point of view. As a consequence, present findings could rely on poly-synaptic and task-dependent networks, characterized by state-dependent, flexible bindings. Thus, different regions, connections and networks could be highlighted if different paradigms were applied (e.g., Bestmann et al., 2008). Indeed, during motor activity or cognitive tasks, PMd could be differently connected with other brain areas, resulting in networks that are different from what has been here observed. Thus, the present work might have revealed one of the possible, task-dependent and long-range networks related with PMd activation.

Finally, the possible spatial limitations of the EEG and the fact that the reconstruction of EEG sources is an inferential process, based on assumptions and a 3D model of the conductive volumes for solving the ill-posed inverse problem should be considered (Bai et al., 2007).

## Conclusions

In conclusion, the present study corroborates and extends previous findings on the connectivity of PMd. In particular, our results shed light on the late temporal dynamics and connectivity among left PMd and mainly contralateral and posterior regions of the brain in right handed healthy humans.

### Conflict of interest statement

The authors declare that the research was conducted in the absence of any commercial or financial relationships that could be construed as a potential conflict of interest.
